# Characterization and Cytotoxicity of *Pseudomonas* Mediated Rhamnolipids Against Breast Cancer MDA-MB-231 Cell Line

**DOI:** 10.3389/fbioe.2021.761266

**Published:** 2021-11-30

**Authors:** Neelam Mishra, Kavita Rana, Siva Deepthi Seelam, Rakesh Kumar, Vijyendra Pandey, Bharathi P. Salimath, Dayanand Agsar

**Affiliations:** ^1^ Department of Microbiology, Gulbarga University, Gulbarga, India; ^2^ Department of Toxicology, Chaudhary Charan Singh University, Meerut, India; ^3^ Department of Life Science, School of Life Sciences, Central University of Karnataka, Kadaganchi, India; ^4^ Department of Psychology, School of Social and Behavioural Sciences, Central University of Karnataka, Kadaganchi, India; ^5^ Department of Biotechnology, University of Mysore, Mysore, India; ^6^ Sanorva Biotech Pvt. Ltd., Mysuru, India

**Keywords:** MDA-MB-231 cell lines, rhamnolipid, MTT, trypan blue, p38MAPK, TNBC, resazurin, cytotoxic

## Abstract

A biosurfactant producing bacterium was identified as *Pseudomonas aeruginosa* DNM50 based on molecular characterization (NCBI accession no. MK351591). Structural characterization using MALDI-TOF revealed the presence of 12 different congeners of rhamnolipid such as Rha-C8-C8:1, Rha-C10-C8:1, Rha-C10-C10, Rha-C10-C12:1, Rha-C16:1, Rha-C16, Rha-C17:1, Rha-Rha-C10:1-C10:1, Rha-Rha-C10-C12, Rha-Rha-C10-C8, Rha-Rha-C10-C8:1, and Rha-Rha-C8-C8. The radical scavenging activity of rhamnolipid (DNM50RL) was determined by 2, 3-diphenyl-1-picrylhydrazyl (DPPH) assay which showed an IC_50_ value of 101.8 μg/ ml. The cytotoxic activity was investigated against MDA-MB-231 breast cancer cell line by MTT (4,5-dimethylthiazol-2-yl-2,5-diphenyl tetrazolium bromide) assay which showed a very low IC50 of 0.05 μg/ ml at 72 h of treatment. Further, its activity was confirmed by resazurin and trypan blue assay with IC_50_ values of 0.01 μg/ml and 0.64 μg/ ml at 72 h of treatment, respectively. Thus, the DNM50RL would play a vital role in the treatment of breast cancer targeting inhibition of p38MAPK.

## Introduction

Biosurfactants are a group of amphipathic compounds that moderate the surface and interfacial tension of liquids (De almeida et al., 2016; Gudiña et al., 2016). They harbor saturated and unsaturated fatty acids as hydrophobic moieties along with polysaccharides, peptides, amino acids, and anions/cations as hydrophilic moieties. There are different groups of biosurfactants, out of which glycolipids are the most preferred due to their low molecular weight (Banat et al., 2014a). Also, they can be synthesized using hydrocarbons, agro-industrial waste, frying and olive oil wastes (Inès and Dhouha, 2015). Rhamnolipids (RLs) are the extensively studied glycolipid based biosurfactant due to its properties such as low critical micellar concentration (CMC: 10–200 mg/ L), surface tension reduction (up to 28–31 mN/ m), high emulsification index (60–70%), and high production in a short period of time (Gudiña et al., 2016).

Different microorganisms can produce different congeners or homologs of RLs by using a variety of sugars, hydrocarbons, and other oil/dairy product wastes (Shao et al., 2017; Pathania and Jana, 2020). *Pseudomonas aeruginosa*, a predominant microbial species, can produce various RL congeners with the most common ones as Rha-Rha-C10-C10, Rha-C10, Rha-C10-C10, and Rha-Rha-C10 (Hošková et al., 2015). RLs have gained importance in various industries such as food, healthcare, pharmaceuticals, and petrochemicals due to their wide range of properties along with enhanced oil recovery, biodegradation, and bioremediation. RLs are known to inhibit proliferation of cancer cells by inducing apoptosis. They regulate humoral and cellular immune response by acting as immunomodulators. They act as antimicrobial agents as they can lower the surface hydrophobicity, thus destroying the cytoplasmic membrane causing killing of bacterial cells. They are also involved in synthesis of nanoparticles. Thus, RLs have high biological applications because of its antimicrobial, anticancer, antioxidant, immunomodulatory property and their capability of nanoparticles synthesis (Banat et al., 2014b; Thakur et al., 2021).

Rhamnolipid is recovered from cell free culture broth by solvent extraction or acid precipitation method in partially purified form. Structural elucidation of any surface-active compound is of utmost importance and cannot be confirmed merely with TLC and FTIR as both of them carries some drawbacks and hence it should be accompanied by other methods such HPLC-MS/UPLC-MS and NMR spectroscopy (Twigg et al., 2020). MALDI-TOF is the emerging technology and can be used to characterize biosurfactants (Price et al., 2009). As RLs are composed of mono-RLs and di-RLs along with different congeners, MALDI-TOF has emerged as the screening strategy where new congeners can be determined at intimal stages (Sato et al., 2019).

Breast cancer is one of the most prominent malignant diseases in the world. Worldwide, there are 3,465,951 incidences and 1,121,413 deaths respectively, according to GLOBOCAN 2020. In 2020, 1,204,532 new cases and 436,417 deaths were recorded in India (Sung et al., 2021). Breast cancer affecting the female population continues to increase annually and there is no ideal treatment for the disease (Wu et al., 2017). Doxorubicin, etoposide, and mitoxantrone are some of the widely prescribed anticancer drugs. One of the major causes of high mortality in people with cancer is resistance developed to the classical chemotherapeutic agent, which is being considered to be a major hindrance, leading to major obstructions in cancer treatment (Housman et al., 2014; Wambang et al., 2016; Wang et al., 2019). Besides this, the limited solubility, stability, bio-distribution, and cytotoxic effect on normal cells significantly reduces the effectiveness of chemotherapeutic drugs. Thus, it is essential to create efficient alternative formulations, selectively targeting cancer cells without causing substantial harm to healthy cells (Haque et al., 2021). The life expectancy of cancer patients would be significantly influenced by the development of new chemotherapeutic/chemical substances/agents. RLs have recently been discovered as a promising antitumor agent interfering with the proliferation of cancer cells of different origins (Chen et al., 2017).

Rhamnolipids have shown a significant effect on human as well as animal cancer cells in the recent past. It is reported that di-RLs produced from *Pseudomonas aeruginosa* B189 inhibit the proliferation of a breast cancer cell line MCF-7 and an insect cell line C6/36 (Thanomsub et al., 2006). This anticancer property of RLs was further tested on various cell lines such as BV-173, SKW-3, HL-60, and JMSU-1 derived from tumors of different origin with IC_50_ values of 50–140 µM (Christova et al., 2013). RLs isolated from *Pseudomonas aeruginosa* MR01 inhibited HeLa cancer cells growth at the concentration of 5 μg/ ml (Loftabad et al., 2010). Burgos-Díaz et al. (2013) reported anticancer activity of glycolipid from *Sphinobacterium detergens* against colorectal cancer cell line. Trehalose lipid, a glycolipid from *Mycobateria,* was used as an antitumor agent (Fracchia et al., 2015).

Similarly, Rahimi et al. (2019) investigated the cytotoxic effect of RL-1 and RL-2 isolated from *Pseudomonas aeruginosa* MR01 against MCF-7, a breast cancer cell line. The results clearly indicate the antiproliferative effect of RLs, emphasizing induction of apoptosis. They reported increased expression of p53 gene in mRNA levels, indicating induction of cell cycle control by RLs in cancer cells. All these reports give insight into the use of RLs as a potential therapeutic antitumor agent.

TNBC, commonly referred to as triple negative breast cancer cell line, lacks expression of ER−a estrogen receptor, PR−a progesterone receptor, and HER 2-a human epidermal growth factor receptor 2 (Chavez et al., 2010). Poor long-term outcomes are associated with it, as compared to other breast cancer (Li et al., 2017). The MDA-MB-231 cell line also lacks expression of these receptors and thus is an aggressive and invasive breast cancer cell line (Cailleau et al., 1978). It is insensitive to anti-hormone-based therapies, including tamoxifen due, to the absence of ER expression (Osborne and Schiff, 2011). It accounts for approximately 15% of breast cancer diagnosed worldwide, which amounts to almost 200,000 cases each year. It is more commonly diagnosed in women younger than 40 years, compared with hormone receptor-positive breast cancer (Swain, 2008). These types of breast cancer have limited treatment options and thus it is commonly used for development of novel therapeutic approaches by exploring molecular basis of this type of breast cancer (Mohammed et al., 2020).

The involvement of p38MAPK signaling pathway, a group of stress-activated kinases in apoptotic cell death phenomenon, makes this pathway striking for cancer researchers (García-Cano et al., 2016). The inhibition of p38MAPK plays a remarkable role in cancer therapy. P38MAPK is a crucial player in response to chemotherapy, as apoptosis is the main mechanisms associated with it (Sanchez-Prieto et al., 2000; Cai et al., 2006). In few cases of colorectal cancer, experimental evidence supports the fact that p38MAPK inhibition by itself is a promising target in cancer therapy (Gupta et al., 2015; Igea and Nebreda 2015). In primary acute myeloid leukemia, the p38MAPK inhibition overcomes the resistance to compounds such as Birinpant (Lalaoui et al., 2016). Ongoing trials showed these inhibitors safety for ralimetinib and ARRY-614 (Garcia-Manero et al., 2015; Patnaik et al., 2016). The implication of active p38MAPK in cancer is not a genetic alteration in the MAPK, instead a pathological context of a tumor. Thus, it is advisable to search for P38MAPK inhibitor for cancer researchers (Pritchard and Hayward 2013). Hence, the use of p38MAPK can be considered as a potential target for cancer therapy. A new era of better prognosis and personalized treatment can be led by a deep understanding of the role of p38MAPK in cancer therapy.

The DNM50RL (a mixture of mono-RL and di-RL congeners) used in the present study was produced using *Pseudomonas aeruginosa* DNM50. MALDI-TOF was used to characterize RLs in view to identify its different congeners. Radical scavenging activity was assessed though DPPH assay. Cytotoxic effect of DNM50RL was assessed against MDA-MB-231 triple negative cell line through MTT, Resazurin, and Trypan blue assay. Since p38 plays a crucial role in anticancer therapy, inhibition of phosphorylated p38 was examined using Western blot analysis.

## Materials and Methods

### Chemicals Used

Chemicals and media used in the present study were obtained from Himedia (Mumbai, India), Sigma Aldrich Pvt. Ltd. (United States), and Merck and Co. Inc. (United States).

### Characterization of Microbial Strain

*Pseudomonas aeruginosa* DNM50 used in the present study was previously isolated from oil contaminated soil sourced from railway tracks of Wadi, Kalaburagi, Karnataka, India, in A-DBT Research Laboratory, Gulbarga University, Kalaburagi. Seven different screening methods were used to confirm its efficiency for biosurfactant production, and the data was reported in our earlier study (Mishra et al., 2021).

*Pseudomonas aeruginosa* DNM50 was identified and characterized using 16 S rRNA sequencing. Spin column kit (Qiagen, Hilden, Germany) was used to extract chromosomal DNA while purification of bacterial 16 S rRNA (1500) was done by using exonuclease I-Shrimp Alkaline phosphatase (Exo-SAP) (Darby et al., 2005). For the amplification of 200 ng/ µL of 16 S rRNA genes, PCR technique was applied using universal primers, 27F (AGA​GTT​TGA​TC(C/A)TGG​CTC​AG) and 1492R (TACGG (C/T) TACCTTGTTACGACTT) (Clarridge 2004; Mulla et al., 2016; 2018). Amplicons of PCR amplified genes were sequenced by following the method of Sanger (Sanger et al., 2000) using ABI 3500xL genetic analyzer (Life Technologies, United States). Sequencing files (.ab1 format) were edited with CHROMASLITE (version1.5) and then analyzed with the Basic Local Alignment Search Tool (BLAST) against the closest culture sequence retrieved from the National Centre for Biotechnology Information (NCBI) database, which identifies regions of local similarity between sequences. The evolutionary history and evolutionary analysis were deduced by Neighbor-Joining method and MEGA 6, respectively (Altschul et al., 1990; Tamura et al., 2013).

### Characterization of Rhamnolipids

The RLs produced by *Pseudomonas aeruginosa* DNM50 under submerged process was subjected for its chemical characterization. The production medium used was neem oil cake extract (19.5%), as a sole source of nutrition. The extract of neem oil cake (pH 7.5) was prepared (Amena et al., 2010), sterilized and inoculated with 10% of 24 h culture followed by incubation at 40°C with agitation of 200 rpm for 5 days. Growth stages of the culture, production levels of RL, and surface tension (SFT) reduction of the production medium were recorded for a period of 5 days. All experiments were carried out in triplicates. The mean values were plotted along with standard deviation (Hazra et al., 2010; Samykannu and Achary, 2017; Chopra et al., 2020).

Cell free broth was used for extraction of RLs as per the modified procedure described by Chandankere et al. (2013). After 3 days of fermentation, culture broth was centrifuged for 10 min at 10,000 rpm. The cell free broth was filtered through 0.45 µM filter and extracted three times with equal volume of chloroform. The organic phase was collected, and solvent evaporated using a rotavapour. The resulting brownish semisolid compound was dried in an oven at 70°C and subjected to TLC (compared with reference standard of RLs from AGAE technologies, USA). The extracted RL (DNM50RL) was further analyzed by HPLC (not reported here) and MALDI—TOF.

#### Thin Layer Chromatography

For thin layer chromatography, a sample of DNM50RL was dissolved in chloroform and 20 µL of the aliquot was applied to pre-coated silica gel (F 1500 LS. 254; Schleicher-Schull, Germany). A mixture of butanol:acetic acid:water in the ratio of 2:1:1 (v/v/v) was used as the mobile phase. Molisch reagent (α-naphthol in ethanol with 10% H_2_SO_4_), ninhydrin reagent, and iodine vapors were used for detection of carbohydrate, proteins, and lipids, respectively (Loftabad et al., 2010).

#### Matrix Assisted Laser Desorption/Ionization Time of Flight

To elucidate the structure of RLs, MALDI-TOF was performed using an Applied Biosystem Ultraflextreme, Bruker Daltonic Germany Mass Spectrometer in reflection mode (Maiqian et al., 2010).

### Antioxidant Activity

#### DPPH Assay

The 2,2,-diphenyl-1-picrylhydrazyl (DPPH) free radical scavenging potential of DNM50RL was examined according to the method described by Li et al. (2014). A concentration range of 0.00625–5 mg/ml was used for both DNM50RL and ascorbic acid (standard). The absorbance at 517 nm determines the reduction of DPPH radical and the calculation for radical scavenging activity was done as follows,

DPPH radical scavenging % = [(A_0_-A_1_)/A_0_] x 100. where DPPH absorbance is denoted by A_0_ and absorbance of sample is denoted by A_1_.

The antioxidant property is measured in % radical scavenging activity and the results are presented as the average of three independent experiments.

The IC_50_ (effective concentration to scavenge DPPH radical) value of DNM50RL was determined by GraphPad prism software version 9.1.0.221. The dose response curve is plotted between DPPH radical scavenging activity and the concentration.

### Cytotoxic Activity

#### Cell Cultures

MDA-MB-231, a tumorigenic, invasive, metastatic, and TNBC representative cell line was procured and authenticated from National Centre for Cell Sciences (NCCS), Pune, India. The cells were cultured in L-15 (Leibovitz’s 15) medium with 2 mM L-glutamine and 10% FBS (fetal bovine serum) and were maintained at 37°C.

#### MTT Assay

5 × 10^4^ cells (MDA-MB-231) were cultured in 96-well plate (Nunc MicroWell™) in complete Dulbecco’s Modified Eagle Medium (DMEM) medium and incubated overnight at 37°C in 5% CO_2_. Cells were exposed to different concentrations (0.01, 0.05, 0.1, 0.5, 1.0, and 5.0 μg/ ml) of compound in triplicates with vehicle control (0.05% DMSO served as negative control) and positive control (Etoposide in the concentration range of 1—1000 µM). 24 h of post-treatment, 10 μL of 4,5-dimethylthiazol-2-yl-2,5-diphenyltetrazolium bromide (MTT) (5 mg/ ml) was added in each well and incubated for 4 h at 37°C. The resultant purple formazan crystals were solubilized in 150 μL of DMSO. The color development was recorded at a test wavelength of 570 nm and a reference wavelength of 630 nm (Varioskan™ Flash Multimode Reader, Thermo Scientific, Switzerland). The cytotoxicity in percentage was derived against control cells as 100% (Riss et al., 2016; Byrappa et al., 2017). The cytotoxicity measurements were depicted in the dose-response curve for a period of 24, 48, and 72 h. The IC_50_ value (half-maximal inhibition concentration) was determined using GraphPad Prism software version 9.1.0.221 using nonlinear regression (curve fit).

#### Resazurin Assay

5 × 10^4^ MDA-MB-231 cells were grown in 96-well plate (Nunc MicroWell™) in complete L-15 medium and incubated overnight at 37°C. Cells were treated with increasing concentrations (0.01, 0.05, 0.1, 0.5, 1.0, and 5.0 μg/ml) of the compound in triplicates with vehicle control (0.05% DMSO served as negative control) and a positive control (Etoposide in the concentration range of 1—1000 µM). After 24 h of treatment, cells were incubated with 100 μL of resazurin sodium salt (0.01 g/2 ml sterile PBS—phosphate buffered saline) at 37°C for 4 h. The resultant change from blue to pink color indicates the presence of viable cells. The optical density of the colored solution was determined at a reference wavelength of 630 nm and test wavelength of 570 nm (Infinite Pro Tecan Multimode reader, Thermo Scientific, Finland). The percentage of cytotoxicity was calculated against control cells as 100% (Riss et al., 2016; Byrappa et al., 2017). The cytotoxicity measurements were analyzed in the dose-response curve for 24, 48, and 72 h. The IC_50_ value (half-maximal inhibition concentration) was demonstrated using GraphPad Prism software version 9.1.0.221 using nonlinear regression (curve fit).

#### Trypan Blue Assay

5×10^4^ MDA-MB-231 cells were seeded in 6-well plate in complete L-15 medium and cells were treated with increasing concentrations (0.01, 0.05, 0.1, 0.5, 1.0, and 5.0 μg/ ml) of the compound with control. After 24 h of treatment, cells were stained with trypan blue (0.4% trypan blue in PBS—phosphate buffered saline, pH 7.2–7.3). A mixture of treated cells (10 µL), trypan blue (10 µL), and PBS (980 µL) were resuspended and 10 µL of the mixture was loaded from the edge of a coverslip placed on a hemocytometer. The number of viable cells/mL was enumerated by using the following formula.

Average of cells counted from four squares x 10,000 x dilution factor (i.e., 100)

= Average cells count x 10^6^ cells.

Cell viability (%) = (Number of live cells ÷ Number of total cells) × 100.

Three independent readings were taken. All the measurements were depicted in the dose-response curve for 24, 48, and 72 h (Phillips, 1973). Etoposide in the concentration range of 1–1000 µM served as the positive control along with 0.05% DMSO as negative control. The IC_50_ value (half-maximal inhibition concentration) was determined using GraphPad Prism software version 9.1.0.221 using nonlinear regression (curve fit).

### Western Blotting for Regulation of P38

MDA-MB-231 cells were revived and grown until 80% confluent, the cells were trypsinized (trypsin-EDTA 0.25%), split, and then seeded into sterile Petri dishes. After 80–90% confluency, the media was aspirated, and cells were starved overnight with basal media. MDA-MB 231 cells were treated with 2 mM sodium orthovanadate for 2 h, followed by 5 µg of compound for 24, 48, and 72 h. The cell lysate was prepared using chilled lysis RIPA buffer. Cell lysate of 24, 48, and 72 h was collected. After estimating protein (Bradford, 1976), the supernatant was loaded on gel (150 µg/well) and processed for Western blotting using a semidry blotter. The unwanted protein binding sites on the membrane were blocked by incubating membrane in 5% bovine serum albumin (BSA) for 2 h at room temperature with moderate agitation. Blot was washed three times with TBST buffer (tris-buffered saline, 0.1% tween 20) for 10 min each. Membrane was incubated overnight at 4°C with moderate agitation in primary antibody (phosphorylated p38, polyclonal rabbit antibody), diluted as 1:1000 ratio in blocking buffer. After washing the blot, the blot was incubated with secondary goat anti-rabbit HRP tagged antibody in 1:3000 ratio for 2 h at room temperature with gentle agitation. Following washing, the blot was developed using enhanced chemiluminescence (ECL). Blot was stripped and re-probed with total p38 antibody to show equal loading in all blots. β actin served as the loading control. Membrane was washed with TBST extensively and continued with blocking procedure. Quantification of protein bands were determined by ImageJ software (Towbin et al., 1979).

### Statistical Analysis

All the results were expressed as the Mean ± SD values obtained in triplicate from three independent tests. SPSS version 22.0 was used to perform all statistical analysis. One-way ANOVA followed by Tukey’s HSD Post-Hoc test and Student’s t test were used to make multiple comparisons with control groups. *p < 0.05 represents statistically significant values.

## Results and Discussion

### Strain Characterization

**FIGURE 1:**
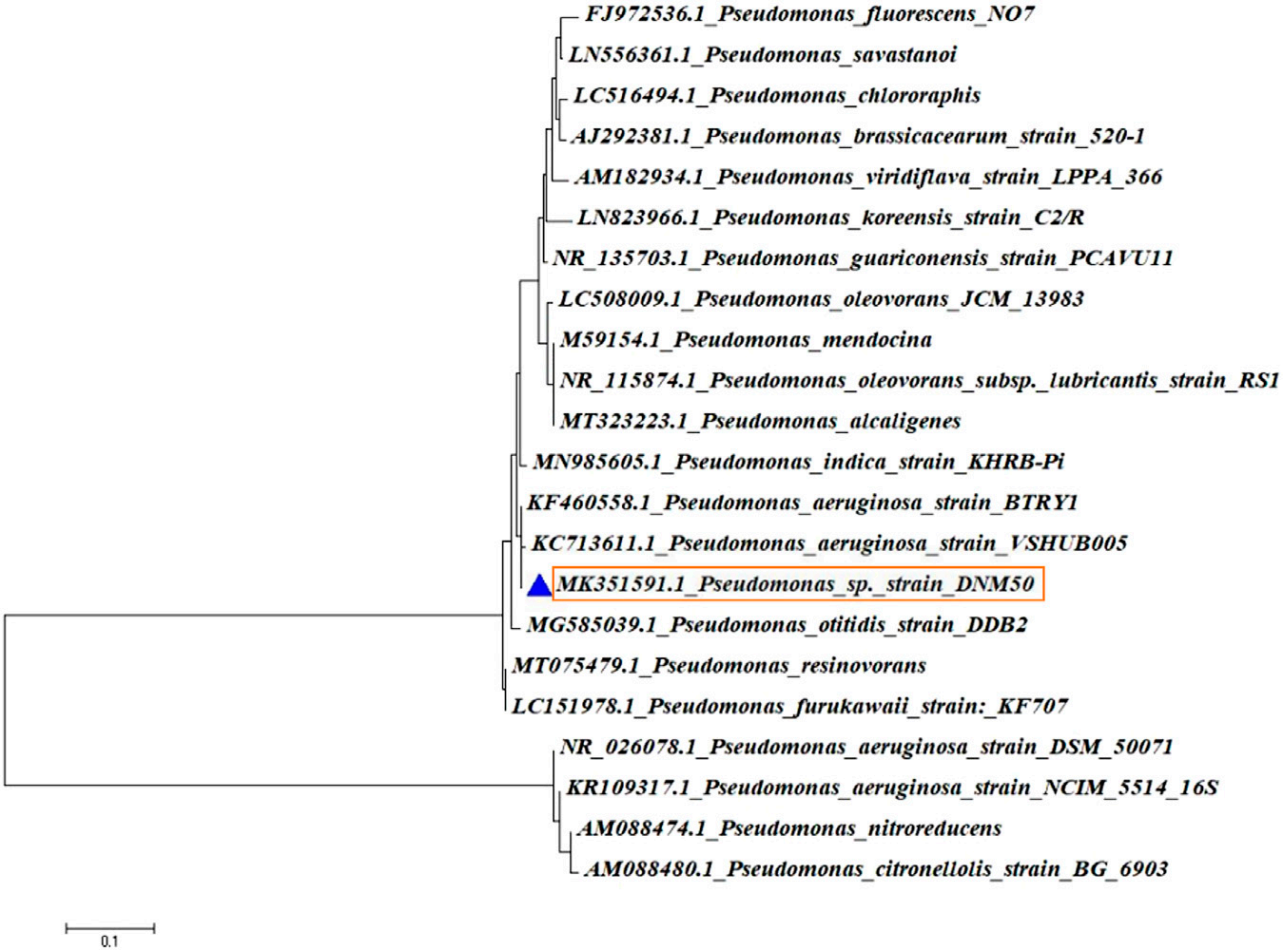
Phylogenetic tree of the isolate DNM50.

The isolated genomic DNA of the potential isolate was amplified to obtain a PCR product, which was further purified. It was then subjected to 16SrRNA gene sequencing and identified by phylogenetic analysis. The consensus sequences available from National Centre for Biotechnology Information (NCBI) were used to perform Nucleotide Blast (Blast-n). Sequence alignment was done using CLUSTAL–W and a phylogenetic tree was constructed using MEGA 6 software as shown in Figure 1. The potential isolate DNM50 was identified based on phylogenetic analysis and designated as *Pseudomonas aeruginosa* DNM50 (99% similarity with *Pseudomonas aeruginosa*). The sequence was submitted to NCBI and the GenBank accession number MK351591 was obtained.

### Characterization of Rhamnolipids

A linear increase in the growth of the culture was observed up to 60 h and was declined further. Maximum production of RLs (2.9 g/ L) was achieved at 72 h with no significant increase up to 120 h. A consistent decrease in SFT (from 62 mN/ m to 30 mN/ m) of the medium was recorded up to 120 h (Supplementary Figure S1).

**FIGURE 2:**
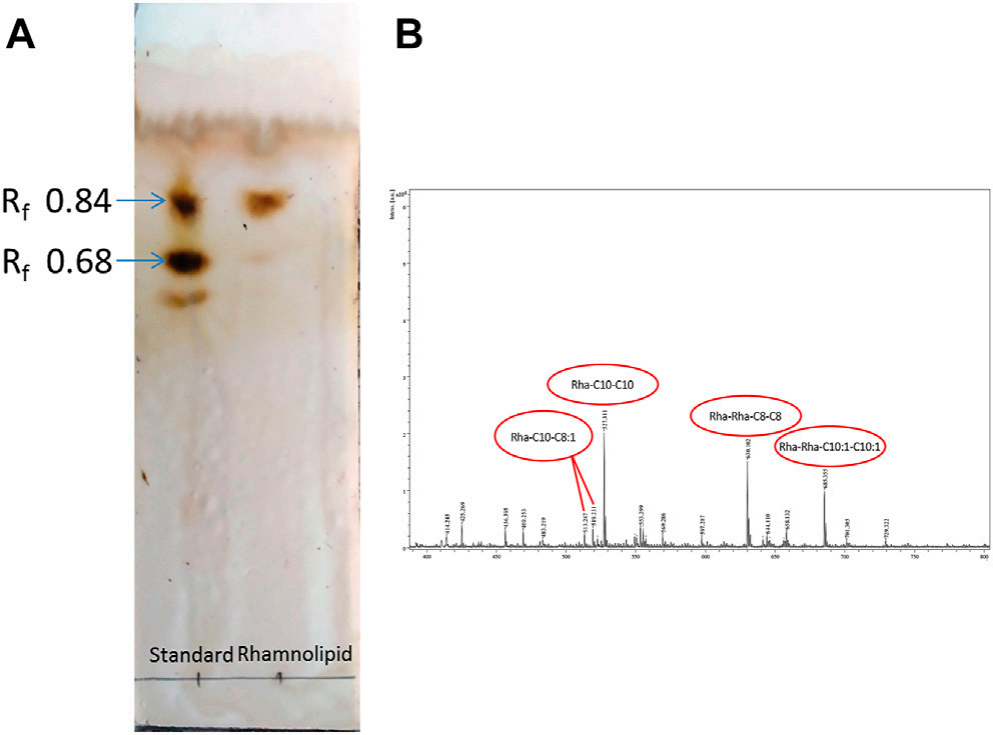
Chemical characterization of Rhamnolipid (DNM50RL) produced from *Pseudomonas aeruginosa* DNM50 by **(A)** thin-layer chromatography (TLC): TLC plate run in a solvent system of butanol:acetic acid:water (2:1:1), developed with Molisch reagent (α—naphthol in ethanol and H_2_SO_4_) showing typical brown color spots of Rhamnolipid. **(B)** MALDI TOF peaks.

Thin layer chromatogram (Figure 2A) of RLs produced by *Pseudomonas aeruginosa* DNM50 showed two characteristic spots corresponding to standard RLs on reaction with Molisch reagent with retardation factor (R_f_) values of 0.84 and 0.68 confirming the presence of mono-RLs and di-RLs, respectively. Positive reaction with Molisch reagent and iodine vapors ratifies glycolipid nature of RLs. No spots were observed with ninhydrin reagent indicating absence of amino acids/proteins. Many researchers reported similar RLs mixture with varying composition (Haba et al., 2003; Silva et al., 2010; Abbasi et al., 2012).

MALDI-TOF (Figure 2B) confirmed the presence of mono-RL and di-RL congeners, specifically Rha-C8-C8:1, Rha-C10-C8:1, Rha-C10-C10, Rha-C10-C12:1, Rha-C16:1, Rha-C16, Rha-C17:1, Rha-Rha-C10:1-C10:1, Rha-Rha-C10-C12, Rha-Rha-C10-C8, Rha-Rha-C10-C8:1, and Rha-Rha-C8-C8 as shown in Table 1. The spectrum was characterized by molecular ions for mono-RLs (m/z 470-575) and di-RLs (m/z 640-730). Major ions at m/z 527 were attributed to [M + Na]^+^ adduct ions of the major mono-RL, Rha-C10-C10 and m/z 630 and 685 were attributed to [M + K]^+^ adduct ions of the major di-RLs, Rha-Rha-C8-C8, and Rha-Rha-C10:1-C10:1, respectively. These findings were in accordance with the work previously reported for analysis of RLs produced by different strains of *Pseudomonas aeruginosa* (Price et al., 2009; Sarachat et al., 2010; Abeer Mohammed et al., 2018).

**TABLE 1:** Molecular ions observed in MALDI-TOF for Rhamnolipids produced by *Pseudomonas aeruginosa* DNM50.

Rhamnolipids	Molecular formula	Calcd mass units [M]	[M + Na]^+^		[M + K]^+^		[M—H +2Na]^+^		Observed
			Obsd	Calcd	Obsd	Calcd	Obsd	Calcd	
Rha-C8-C8:1	C_22_H_38_O_9_	446.2	469	469.2	483-2H	485.2	−	491.2	
Rha-C10-C8:1	C_24_H_42_O_9_	474.2	−	497.2	513	513.2	519	519.2	
Rha-C10-C10	C_26_H_48_O_9_	504.3	527	527.3	−	543.2	−	549.3	
Rha-C10-C12:1	C_28_H_50_O_9_	530.3	553	555.3	569	569.3	−	575.3	
Rha-C16:1	C_22_H_38_O_7_	414.2	−	437.2	−	453.2	−	459.2	414
Rha-C16	C_22_H_40_O_7_	416.2	−	439.2	456 + H	455.2	−	461.2	
Rha-C17:1	C_23_H_40_O_7_	428.2	−	451.2	−	467.2	−	473.2	425
Rha-Rha-C10:1-C10:1	C_32_H_54_O_13_	646.3	−	669.3	685	685.3	−	691.3	
Rha-Rha-C10-C12	C_34_H_62_O_13_	678.4	701	701.4	−	717.3	−	723.4	
Rha-Rha-C10-C8	C_30_H_54_O_13_	622.3	644-H	645.3	−	661.3	−	667.3	
Rha-Rha-C10-C8:1	C_30_H_52_O_13_	620.3	−	643.3	658 -H	659.3	−	665.3	
Rha-Rha-C8-C8	C_28_H_50_O_13_	594.3	−	617.3	630-3H	633.2	−	639.2	597—3H

Based on chromatographic and spectroscopic analyses, it can be inferred that the biosurfactant produced by *Pseudomonas aeruginosa* DNM50 is comprised of congeners of mono-RLs and di-RLs.

### Radical Scavenging Attribute

**FIGURE 3:**
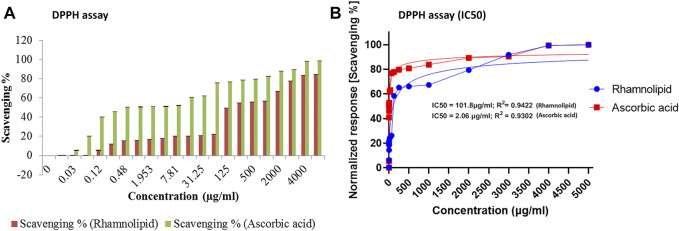
The DPPH radical scavenging activity of DNM50RL and ascorbic acid **(A)** Scavenging %. Values represented as mean ± SD of three independent experiments. Error bars represent SD (*p < 0.05). **(B)** Non-linear regression curve fit (DPPH assay). IC_50_ determined by nonlinear regression (curve fit [Inhibitor] vs. normalized response–Variable slope) using GraphPad prism software.

DPPH assay was developed to examine the antioxidant activity, which relies on the measurement of scavenging ability of antioxidants to a stable free radical, DPPH. Electron of nitrogen atom from DPPH was reduced to corresponding hydrazine by a hydrogen atom from the antioxidant. Reduced form of DPPH shows a color change from deep violet to yellow, thus assessing antioxidant activity of the given compound (Kedare and Singh, 2011). The DPPH radical scavenging activity of DNM50RL and ascorbic acid was as shown in Figures 3A,B.

At 5 mg/ml concentration, DNM50RL and ascorbic acid showed a radical scavenging activity of 84.25 and 98.4%, respectively. This activity increases with an increase in concentration. The IC_50_ value was 101.8 μg/ml for DNM50RL and 2.064 μg/ml for ascorbic acid as determined from GraphPad Prism software. At 1 mg/ml, MCTG107b and MCTG214 (3b1) derived biosurfactant exhibited only 9.67 ± 3.27% and 15.46 ± 4.03% inhibition for DPPH assay (Voulgaridou et al., 2021).

DPPH radical scavenging activity of 69.1 and 73.5% were reported at 5.0 mg/ml of BS-VSG4 and BS-VS16 biosurfactants from *Bacillus* strains (Giri et al., 2019). An IC_50_ value of 4.15 mM for RLs from *Pseudomonas aeruginosa* was deduced (Abdollahi et al., 2020). Similarly, IC_50_ value of 357 μg/ml was observed for DCS1 lipopeptide biosurfactants produced from *Bacillus methylotrophicus* (Jemil et al., 2017). The DNM50RL has shown weak antioxidant activity than standard ascorbic acids but is better than the activity reported by other related studies. The radical scavenging activity for biosurfactants was due to the transfer of protons or electrons, thus neutralizing free radicals. This activity was enhanced by hydrocarbon fatty acid in RLs and was not affected by their variation in chain length (Tofani et al., 2010; Tabbene et al., 2012).

### Cytotoxic Attribute

Cell viability assays such as dye exclusion, colorimetric, fluorometric, luminometric, and flowcytometric are being used by various researchers. However, measurement of cell viability/proliferation cannot be examined by only one method, since it is influenced by types and origins of cell lines and the mechanism of action of the cytotoxic agents explored. Thus, it is recommended to apply more than one assay for cell viability/proliferation (Kamiloglu et al., 2020).

**FIGURE 4:**
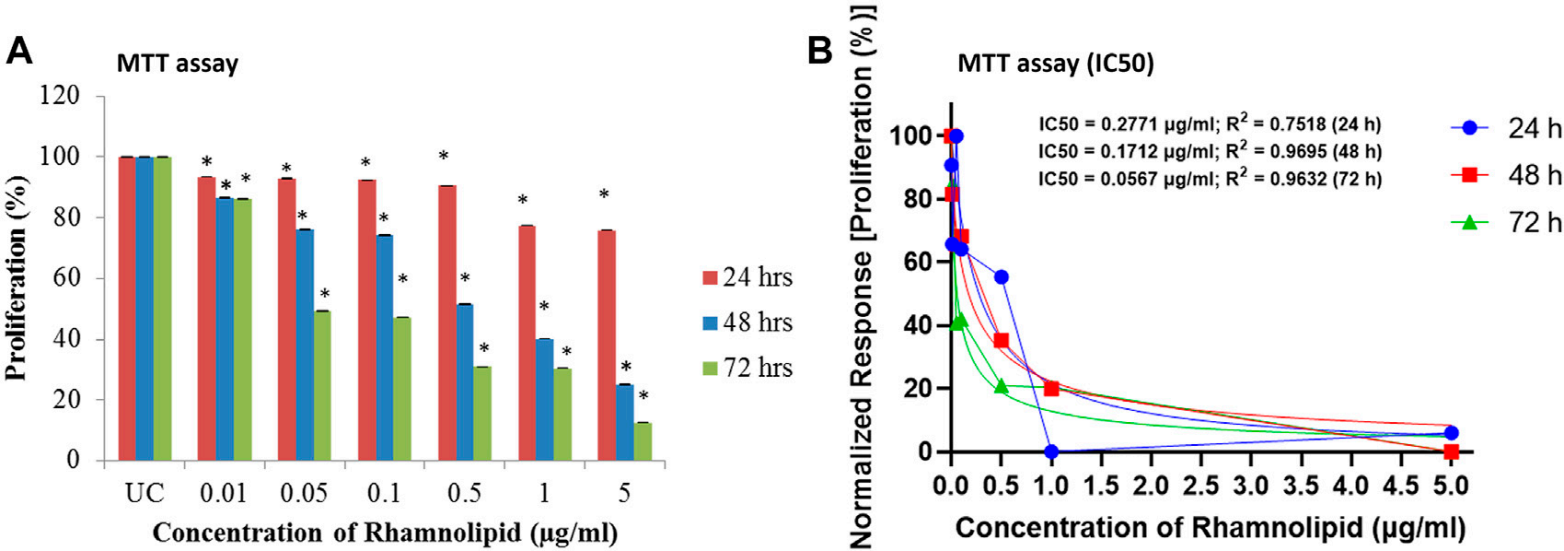
Antiproliferative effect of DNM50RL on MDA-MB 231 cell line by MTT assay after 24, 48, and 72 h of treatment. **(A)** Dose dependent inhibition. Values represented as mean ± SD of three independent experiments. Error bars represent SD (*p < 0.05). **(B)** IC_50_ determination by nonlinear regression [curve fit (Inhibitor) vs. normalized response–Variable slope] using GraphPad prism software.

MTT assay was performed to assess the antiproliferative effect of different concentrations of DNM50RL against MDA-MB-231 cell lines. There is a linear relationship between cell activity and absorbance measuring growth rate of cells, thus MTT is a quantitative and sensitive detection of cell proliferation (Mahajan et al., 2012). MTT can be reduced through the mitochondrial enzyme, which is possible only in the cells with active metabolism. Hence, measurement of cell proliferation is indicated directly by the number of metabolically active/viable cells. With an increase in the concentration of DNM50RL, there is an increase in inhibition of cell proliferation at 24 h. Similar dose-dependent inhibition was observed at 48 and 72 h, with the best results obtained when treatment was given for 72 h showing an IC_50_ value of 0.05 μg/ml (Figures 4A,B).

**FIGURE 5:**
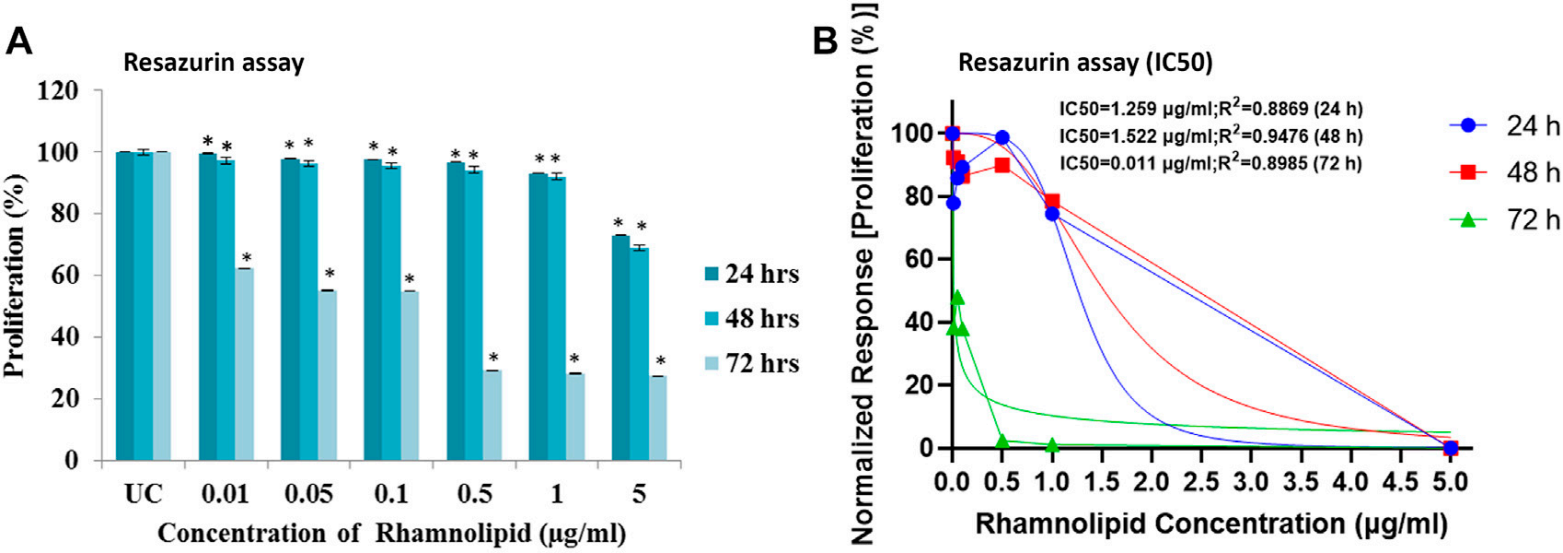
Antiproliferative effect of DNM50RL on human breast cancer cell lines MDA-MB- 231 cell line by resazurin assay after 24, 48, and 72 h of treatment. **(A)** Dose dependent inhibition. Values represented as mean ± SD of three independent experiments. Error bars represent SD (*p < 0.05). **(B)** IC_50_ determined by nonlinear regression [curve fit (Inhibitor) vs. normalized response–Variable slope] using GraphPad prism software.

In resazurin assay, viable cells with active metabolism cause reduction of resazurin to resofurin product, which is pink and fluorescent (Riss et al., 2016). The assay is more sensitive than the MTT assay (Shum et al., 2008), hence it is used to confirm further the antiproliferative activity of DNM50RL against MDA-MB-231 cell lines. A dose dependent trend was observed with IC_50_ value of 1.5 μg/ml at 48 h of treatment, though the best effect was observed at 72 h of treatment with IC_50_ of 0.01 μg/ml (Figures 5A,B).

**FIGURE 6:**
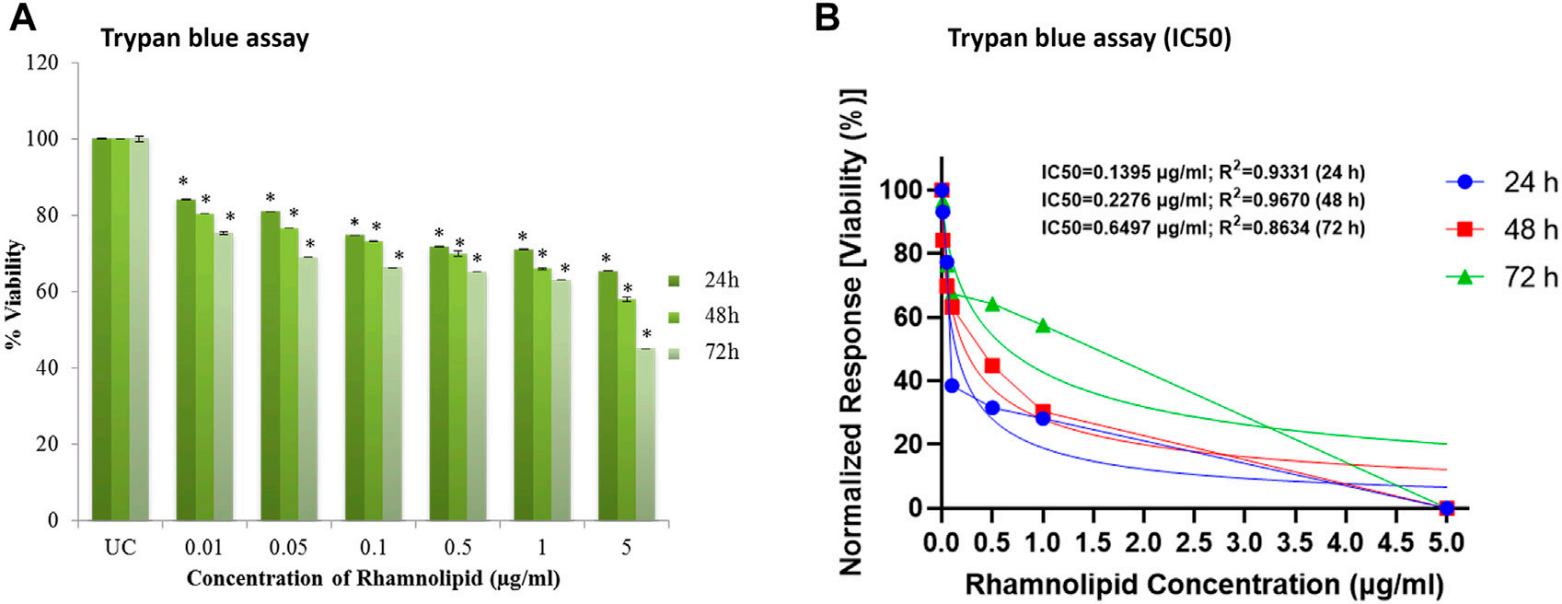
Cell viability efficacy of DNM50RL on human breast cancer cell lines MDA-MB-231 cell line by trypan blue assay after 24, 48, and 72 h of treatment. **(A)** Dose dependent inhibition. Values represented as mean ± SD of three independent experiments. Error bars represent SD (*p < 0.05). **(B)** IC_50_ determined by nonlinear regression [curve fit (Inhibitor) vs. normalized response–Variable slope] using GraphPad prism software.

Trypan blue dye exclusion assay was further used for evaluation of cytotoxicity activity of DNM50RL. This assay easily differentiates live and dead cells as the dead cells take up the dye and live cells exclude trypan blue, hence the name. It is because live cells will retain their membrane intact and dead cells will lose membrane integrity due to apoptosis/necrosis (Farghadani et al., 2017). Figure 6A shows the decrease in cell viability in 72 h by DNM50RL in a dose dependent manner confirming its role in cytotoxicity activity against MDA-MB-231 cell line (Figure 6B). When the treatment was given for 72 h, the IC_50_ value was 0.64 μg/ml. The IC_50_ value of DNM50RL was lower than the positive control, Etoposide (IC_50_ value of 20.65, 25.77, 51.10 µM by MTT, resazurin and Trypan blue assay at 72 h of treatment) (Supplementary Figure S2).

A number of researchers worked on cytotoxic effect of different glycolipid based biosurfactants against different cell lines (Table 2). However, there is limited data available on the cytotoxic effect of RLs against MDA-MB-231 cell line.

**TABLE 2:** IC_50_ values of few glycolipid based biosurfactants against different cell lines.

Biosurfactant	Source	Cell line	Cytotoxic assay	IC50/LC50/GI50/EC50	Treatment time	References
Rhamnolipid 1; Rhamnolipid 2	*Pseudomonas sp.*	HepG2 liver cancer cell line; A549 lung cancer cell line; MDA-MB-231 cell line; HeLa cell line	MTT assay	140 μM, 154 μM, 86 μM, 123 µM; 79 μM, 98 μM, 58 μM, 88 µM	48 h	Kamal et al. (2012)
Glycolipid biosurfactant S9BS	*Lysinibacillus fusiformis* S9	HEK-293, a human embryonic kidney cancer cell	MTT assay	75 μg/ ml (LC50)	24 h	Pradhan et al. (2014)
Monorhamnolipid (NDYS-4E)	*Streptomyces coelicoflavus* NBRC (15399^T^)	MCF-7, a breast cancer cell line	MTT assay	88.60 μg/ ml	48 h	Allada et al. (2015)
BS1a; BS1b	R—95^TM^ RL; R—90 ^TM^ RL	MCF—7, a breast cancer cell line	MTT assay	153.40 μg/ ml,98.27 μg/ ml,33.08 μg/ ml; 168.50 μg/ ml, 42.85 μg/ ml,30.05 μg/ ml	24 h, 48 h, 72 h	Akiyode et al. (2016)
Rhizoleucinoside, a RL - amino-alcohol hybrid	*Bradyrhizobium sp*. BRAil	Murine microglia; Rat microglia	CellTiter-Glo 2.0 (CTG 2.0) assay	6.9 µM; 22 µM	24 h	Chen et al. (2016)
Biosurfactant	*Lactobacillus casei*	HEp-2 cell line	MTT assay	109.1 ± 0.84 mg/ ml to 129.7 ± 0.52 mg/ ml	48 h	Merghni et al. (2017)
Dokdolipids A, B, C	*Actinoalloteichus hymeniacidonis* 179DD-027	MDA-MB-231 breast cancer cell line	Sulforhodamine (SRB) assay	30.6, 40.4, 25.5 µM (GI50)	48 h	Choi et al. (2019)
Monorhamnolipids; Dirhamnolipids	*Pseudomonas aeruginosa* MR01	MCF-7 breast cancer cell line	MTT assay	25.87 μg/ ml; 31 μg/ ml	48 h	Rahimi et al. (2019)
L-SL (Lactonic form of sophorolipids);	*Starmerella bombicola* MTCC1910	Human lung adenocarcinoma epithelial cell line A549;	MTT assay	50 μg/ ml, 40 μg/ ml, 880 μg/ ml, 400 μg/ ml;	24 h, 48 h	Haque et al. (2021)
Glucolipids	*Candida bombicola*	MDA-MB-231 breast cancer cell line;		50 μg/ ml, 40 μg/ ml, 900 μg/ ml, 400 μg/ ml;		
				40 μg/ml, 35 μg/ml, 600 μg/ml, 100 μg/ml		
MCTG107b (mixture of rhamnolipid analogues); MCTG214 (3b1) (dirhamnolipids congeners)	*Marinobacter* strain *Pseudomonas* strain	Mouse skin melanoma cell line B16F10 HaCat keratinocyte cell Line THLE3 liver cell line	Almar blue assay	1.3 ± 0.4 mg/ ml, 0.73 ± 0.1 mg/ ml, 0.76 ± 0.1 mg/ ml (EC50);	24, 48, 72 h	Voulgaridou et al. (2021)
				1.28 mg/ ml±0.2, 1.04 ± 0.1 mg/ ml, 0.96 ± 0.1 mg/ ml (EC50);		
				1.35 ± 0.1 mg/ ml, 0.78 ± 0.1 mg/ ml, 0.55 ± 0.1 mg/ml (EC50);		
				2.3 ± 0.2 mg/ml, 0.99 ± 0.1 mg/ml, 0.84 ± 0.1 mg/ ml (EC50);		
RL1; RL2	*Pseudomonas aeruginosa* BN10	MCF-7, breast cancer cell line;	MTT assay	8.68 μg/ ml, 8.67 μg/ ml;	48 h	Semkova et al. (2021)
		MDA-MB-231 breast cancer cell lines		6.99 μg/ ml, 8.60 μg/ ml		
DNM50RL (a mixture of mono and dirhamnolipids)	*Pseudomonas aeruginosa* DNM50	MDA-MB-231 breast cancer cell line	MTT assay Resazurin assay Trypan blue assay	0.27 μg/ ml.,0.17 μg/ ml,0.05 μg/ ml	24, 48, 72 h	Present study
				1.25 μg/ ml, 1.52 μg/ ml, 0.01 μg/ ml		
				0.13 μg/ ml,0.22 μg/ ml,0.64 μg/ ml		
Etoposide	Standard anticancer drug	MDA-MB-231 cell line	MTT assay, Resazurin assay Trypan blue assay	46.59, 25.22, 20.65 µM	24h, 48h, 72 h	Present study
				88.32, 60.94, 25.77 µM		
				70.25, 56.01, 51.10 µM		

It can be inferred from the results of all cell proliferation/viability assays that DNM50RL is inhibiting proliferation of MDA-MB-231 cell line in a dose dependent and time dependent manner. Moreover, few reports showed that mono-RLs are more effective for inhibition of breast cancer cells and some emphasize di-RLs to be more effective (Thanomsub et al., 2006; Jiang et al., 2014; Rahimi et al., 2019). Due to such variations found in the biological potentials of purified mono-RLs and di-RLs from previous reports, the present study was aimed to reveal the biological attributes of natural RLs in their native form. This is because an appropriate proportion/mixture of mono-RLs and di-RLs may have much pronounced activity than the individual congeners. Many researchers reported a lack of cytotoxic activity of RLs against normal human cells, e.g., Vero cells, PBMN-peripheral blood mononuclear cells. This may be due to the presence of more negatively charged functional groups on cancer cells and its variation in fatty acid profiles with respect to normal cells, which facilitates greater endocytosis activity of cancer cells (Gudiña et al., 2013; Dwivedi et al., 2015). Reports from various studies on RLs against different cancerous and normal healthy cells show discrete results. This may be due to the nature of normal and cancerous cells studied, purity and types of RL variants, and the degree of surface tension reduction (Rahimi et al., 2019).

### Downregulation of P38MAPK

**FIGURE 7:**
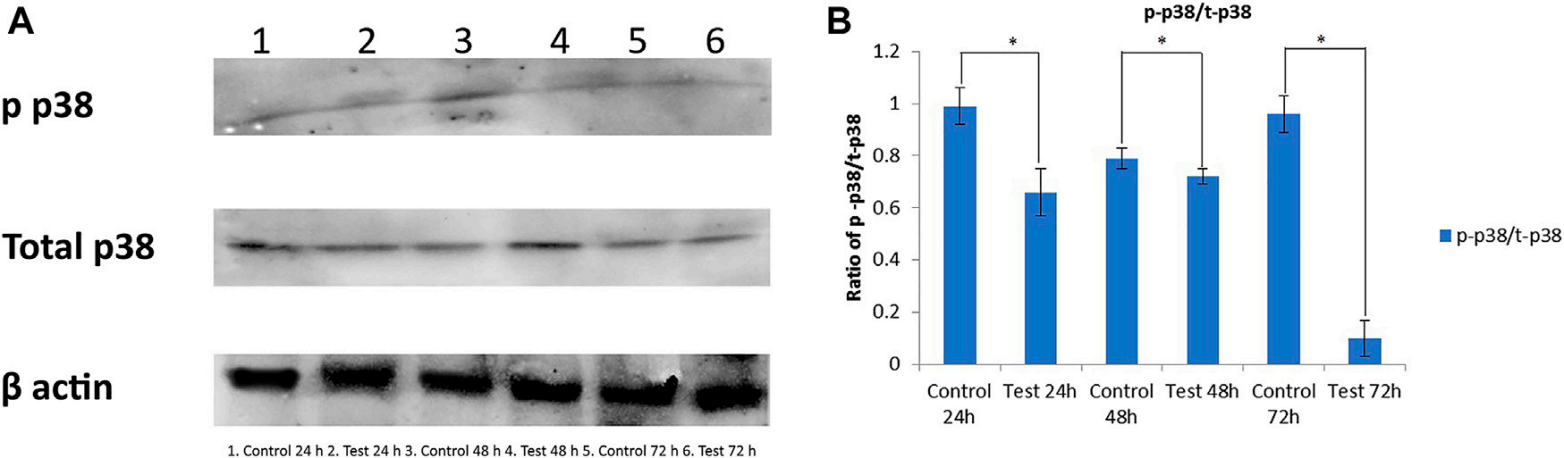
**(A)** Western blot for antibody phosphorylated p38 and total p38. β actin served as loading control. **(B)** Graph representing the ratio of phosphorylated p38 (p-p38)/total p38 (t-p38). The intensity related to expression of p-p38 and t-p38 was analyzed using ImageJ software. All the results are presented as the means ± SD of three independent measurements. Error bars represent standard deviation. (*) Differences were considered significant at *p* < 0.05 as compared with control groups.

Western blot analysis was done to compare the phosphorylated (p-p38MAPK) and total (t-p38MAPK) p38MAPK protein levels after treatment with DNM50RL (5 μg/ ml). This was done to determine whether p38MAPK is responsible for DNM50RL cytotoxicity in triple negative breast cancer cell line MDA-MB-231. As shown in Figures 7A,B, the protein levels of total p38 were higher in these cells until 72 h in control and treated cells (lower panel) while the phosphorylated p38 was downregulated (upper panel).

Several studies showed that p38 mitogen activated protein kinases (MAPKs) play a critical role in cellular responses, proliferation, survival, cell cycle, and migration in cancer. As DNM50RL can downregulate p38 phosphorylation, it can be proposed as a novel and potent anticancer agent with its effectiveness at a very low concentration. RL a (Rha-Rha-C10-C10) isolated from *Pseudomonas aeruginosa* B189 inhibited the proliferation of MCF-7 cells, human breast cancer cell lines with a MIC of 6.25 μg/ml, whereas RL b (Rha-Rha-C10-C12) did not exhibit significant cytotoxic activity. At 50 μg/ml, Rha-Rha-C10-C10 or Rha-Rha-C10-C12 did not show cytotoxic effect against Vero cells, a normal cell line. Thus, this report demonstrates the toxic sensitivity of different congeners of these RLs to other cell lines used (Thanomsub et al., 2006).

Di-rhamnolipids (Rha-Rha-C10-C10 as a major component) produced by *Pseudomonas aeruginosa* M14808 showed antiproliferative efficiency against MCF-7 and H460, a human non-small lung cancer line with a MIC of 1 μg/ml and 5 μg/ml, whereas mono-RLs (Rha-C10-C10 as a major component) and crude extract did not show any antiproliferative activity (Zhao et al., 2013).

Similar sensitivity of RLs (mono-RLs and di-RLs) against two cancer cell lines, HepG2 (a human hepatocellular carcinoma cell line), Caco-2 (a human colon carcinoma cell line), and a normal cell HK-2 (a human proximal tubular epithelial cell line) were reported (Jiang et al., 2014). Mono-RLs presented higher cytotoxicity than di-RLs, which is due to large surface tension reduction by mono-RLs than di-RLs. They stated that similar sensitivity was observed for normal as well as cancerous cell lines with or without fetal bovine serum (FBS). Addition of FBS attenuates the cytotoxic effect of RLs. These observations revealed that RLs reduce surface tension of the culture medium, eliciting its cytotoxic effect, instead of causing any modification in molecular structure. Further, mainly decreased cytotoxic effect was observed *in vivo* as RLs get dissolved in simulated gastric juice. Thus, further research to illustrate high level of biosafety of RLs *in vivo* offers valuable suggestions for cancer researchers (Jiang et al., 2014).

An efficient cytotoxic potency of RLs (Rha-Rha-C10-C10 and Rha-Rha-C10) isolated from *Pseudomonas sp*. strain ICTB -745 was reported against different human cancer cell lines, e.g., lung adenocarcinoma, hepatocellular liver carcinoma, breast adenocarcinoma, and cervical cancer cell lines (Kamal et al., 2012). Mono-RLs (Rha-C10-C10, as a major component) from *Pseudomonas aeruginosa* BN10 had noticeable cytotoxic activity against human urinary bladder carcinoma cell line, human promyelocytic leukemia cell line, human pre-B leukemia cell line, and T-cell chronic lymphocytic leukemia cell line. The probable reason may be the structure of mono-RLs molecule, causing high penetration in tumor cell, leading to cell viability reduction. Mono-RLs induced cell death, are mediated by induction of apoptosis, inactivation of cellular oncogenes or activation/alteration of tumor suppressor process. The dose dependent apoptotic progression of mono-RLs may be due to efflux pump or the receptors present on cell membrane gets saturated, interfering with the permeation of active molecules within the cancerous cells (Christova et al., 2013). *Bcl-2* and *c-myc* gene overexpression in human BV-173 pre-B leukemia cell line were observed with high doses of mono-RLs, resulting in high proliferative and anti-apoptotic effect, which is overcome by prolonged incubation period (≥72 h). Low cytotoxicity against Balb/c 3T3, a non-tumorigenic mouse fibroblast cell line was confirmed by Balb/c 3T3 neutral red uptake test (Christova et al., 2013).

RL-AgNPs from *Pseudomonas aeruginosa* JS-11, at concentration of 10 μg/ ml, showed a remarkable decrease in cell viability of MCF-7 breast cancer cell lines and no significant effect on PBMN—a normal human peripheral blood mononuclear cell was reported. It might be because the anionic metal nanoparticles cause greater attachment into plasma membrane of MCF-7 cells due to the presence of a negatively charged group on the cancer cell surface. This causes greater endocytic activity causing internalization of metal nanoparticles through vesicular transport pathway. Thus, the RL-AgNPs attachment to the cell surface leads to oxidative stress and cell membrane disruption, which assures it as a primary candidate for novel cancer therapy (Dwivedi et al., 2015).

Although many reports are available showing the anticancer activity of the RLs against various other cancer cell lines, there is limited studies available on MDA-MB-231 cell lines. Also, the mechanism of action of RLs at the molecular level is still insufficiently understood. A future study to elucidate the mechanism is needed to learn its biochemical and cellular interaction followed by preclinical studies and clinical trials at different stages (Abbasi et al., 2013; Abalos et al., 2017). The structure and activity relationship studies of pure or mixture of RLs would also offer advantages for researchers to elucidate their biological characterization and predict the biological function of various RL congeners in combination or in particular (Chen et al., 2017).

## Conclusion

Lower toxicity and higher biodegradability attributes of biosurfactants are always advantageous over chemical surfactants for significant biotechnological applications. Isolated and identified strain of *Pseudomonas aeruginosa* DNM50 in the present study is novel due to the significant cytotoxic effect of its RL against triple negative breast cancer cell lines. Twelve different congeners were detected by MALDI-TOF, which contributes to the functional efficiency of RLs. DNM50RL seems to be a potential molecule in the future for cancer therapy due to its very low IC_50_ value against MDA-MB-231 cell line and its significant role in inhibition of p38MAPK. Therefore, the DNM50RL can be a prospective natural, therapeutic anticancer agent.

## Data Availability

The datasets presented in this study can be found in online repositories. The names of the repository/repositories and accession number(s) can be found in the article/Supplementary Material.

## Supplementary Material

Supplementary Figure 1 Growth curve and production of rhamnolipids by Pseudomonas aeruginosa DNM50 in neem oil cake extract as the production medium.

Supplementary Figure 2 Antiproliferative effect of Etoposide (Positive control) on MDA-MB 231 cell lines for 24, 48, and 72 h of treatment by **(A)** MTT assay showing dose dependent inhibition **(B)** IC50 representative graph for MTT assay **(C)** Resazurin assay showing dose dependent inhibition **(D)** IC50 representative graph for Resazurin assay **(E)** Trypan blue assay showing dose dependent inhibition **(F)** IC50 representative graph for Trypan blue assay. For dose dependent inhibition, values are represented as mean ± SD of three independent experiments. Error bars represent SD. Differences with control groups were significant at **p* < 0.05. IC50 was determined by nonlinear (curve fit [Inhibitor] vs. normalized response-Variable slope) using GraphPad prism software.
