# Supplementary figures and images for: Characterization and Cytotoxicity of Pseudomonas Mediated Rhamnolipids Against Breast Cancer MDA-MB-231 Cell Line
Source: Front Bioeng Biotechnol. 2021 Nov 30;9:761266. doi: 10.3389/fbioe.2021.761266 (PMC8691732; doi:10.3389/fbioe.2021.761266)

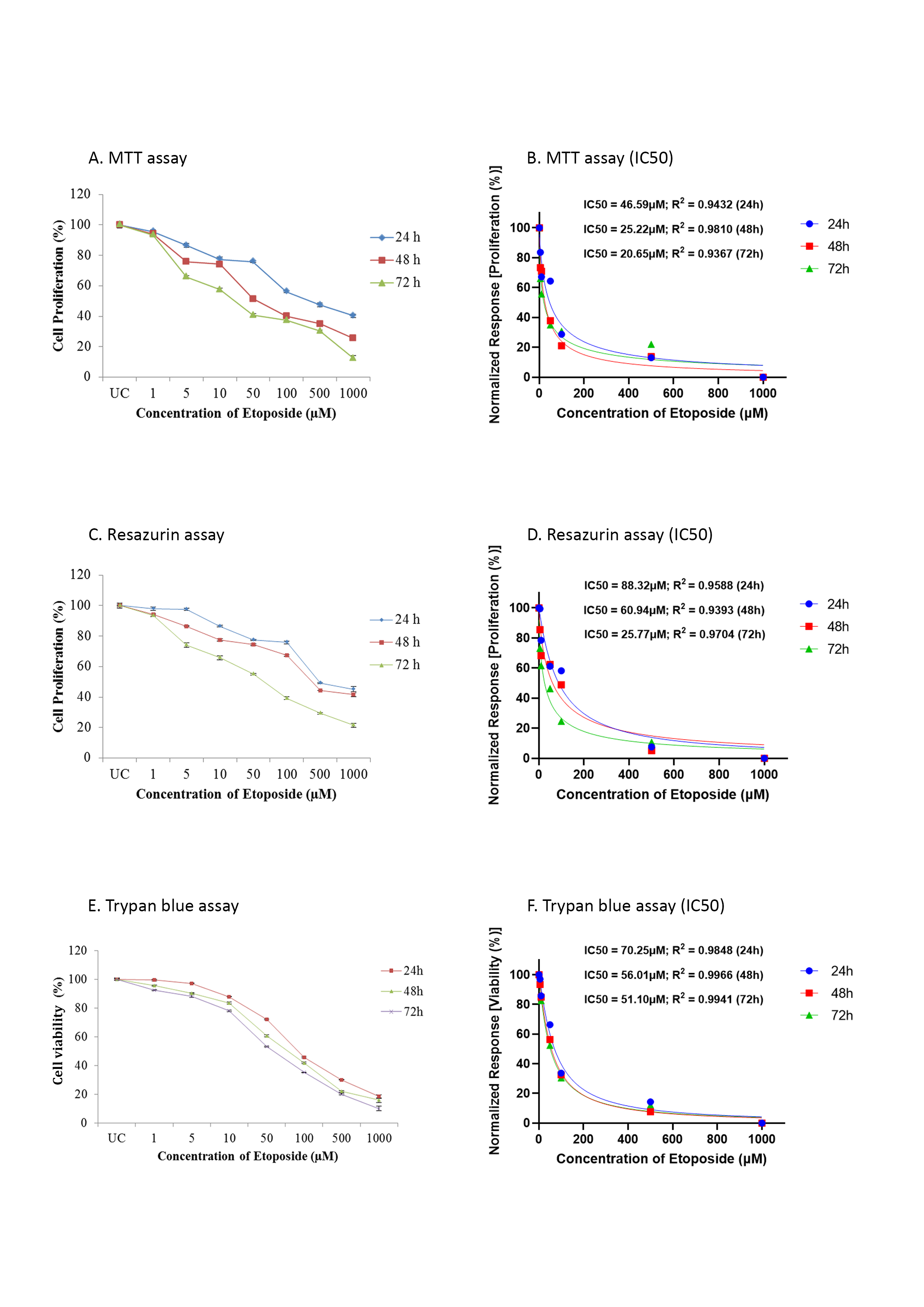

Supplement: Supplementary file 1 [file Image2.jpg]

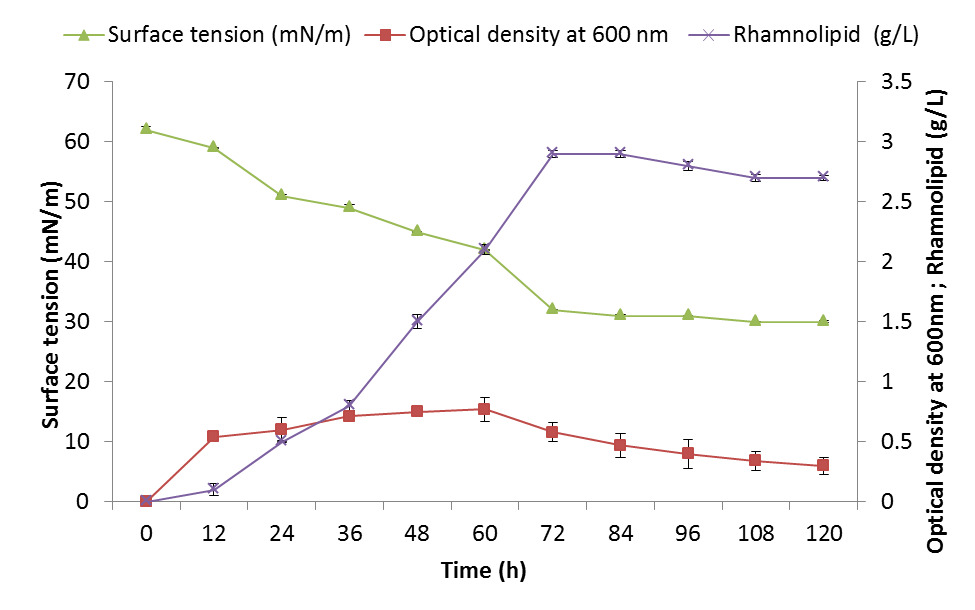

Supplement: Supplementary file 2 [file Image1.jpg]
